# A Two-Time Point Analysis of Gut Microbiota in the General Population of Buenos Aires and Its Variation Due to Preventive and Compulsory Social Isolation During the COVID-19 Pandemic

**DOI:** 10.3389/fmicb.2022.803121

**Published:** 2022-03-24

**Authors:** Pablo Aguilera, María Florencia Mascardi, Fiorella Sabrina Belforte, Ayelén Daiana Rosso, Sofía Quesada, Ignacio Llovet, Gregorio Iraola, Julieta Trinks, Alberto Penas-Steinhardt

**Affiliations:** ^1^Consejo Nacional de Investigaciones Científicas y Técnicas (CONICET), Buenos Aires, Argentina; ^2^Instituto de Medicina Traslacional e Ingeniería Biomédica (IMTIB), CONICET, Instituto Universitario del Hospital Italiano (IUHI), Hospital Italiano de Buenos Aires (HIBA), Buenos Aires, Argentina; ^3^Laboratorio de Genómica Computacional (GEC-UNLu), Departamento de Ciencias Básicas, Universidad Nacional de Luján, Luján, Argentina; ^4^Departamento de Ciencias Básicas, Instituto de Ecología y Desarrollo Sustentable (INEDES) Consejo Nacional de Investigaciones Científicas y Técnicas (CONICET)-UNLu, Universidad Nacional de Luján, Luján, Argentina; ^5^Departamento de Ciencias Sociales, Universidad Nacional de Luján, Luján, Argentina; ^6^Microbial Genomics Laboratory, Institut Pasteur de Montevideo, Montevideo, Uruguay; ^7^Wellcome Sanger Institute, Hinxton, United Kingdom; ^8^Center for Integrative Biology, Universidad Mayor, Santiago de Chile, Chile; ^9^Fundación H.A. Barceló, Instituto Universitario de Ciencias de la Salud, Buenos Aires, Argentina

**Keywords:** COVID-19, gut microbiota, 16S/18S ribosomal RNA gene analysis, Buenos Aires, taxonomic analysis, functional analysis

## Abstract

The COVID-19 pandemic poses a great challenge to global public health. The extraordinary daily use of household disinfectants and cleaning products, social distancing and the loss of everyday situations that allow contact between individuals, have a direct impact on the transfer of microorganisms within the population. Together, these changes, in addition to those that occur in eating habits, can affect the composition and diversity of the gut microbiota. A two-time point analysis of the fecal microbiota of 23 Metropolitan Buenos Aires (BA) inhabitants was carried out, to compare pre-pandemic data and its variation during preventive and compulsory social isolation (PCSI) in 2020. To this end, 23 healthy subjects, who were previously studied by our group in 2016, were recruited for a second time during the COVID-19 pandemic, and stool samples were collected from each subject at each time point (*n* = 46). The hypervariable region V3-V4 of the 16S rRNA gene was high-throughput sequenced. We found significant differences in the estimated number of observed features (*p* < 0.001), Shannon entropy index (*p* = 0.026) and in Faith phylogenetic diversity (*p* < 0.001) between pre-pandemic group (PPG) vs. pandemic group (PG), being significantly lower in the PG. Although no strong change was observed in the core microbiota between the groups in this study, a significant decrease was observed during PCSI in the phylum Verrucomicrobia, which contributes to intestinal health and glucose homeostasis. Microbial community structure (beta diversity) was also compared between PPG and PG. The differences observed in the microbiota structure by unweighted UniFrac PCoA could be explained by six differential abundant genera that were absent during PCSI. Furthermore, putative functional genes prediction using PICRUSt infers a smaller predicted prevalence of genes in the intestinal tryptophan, glycine-betaine, taurine, benzoate degradation, as well as in the synthesis of vitamin B12 during PCSI. This data supports the hypothesis that the microbiome of the inhabitants of BA changed in the context of isolation during PCSI. Therefore, these results could increase the knowledge necessary to propose strategic nutraceutical, functional food, probiotics or similar interventions that contribute to improving public health in the post-pandemic era.

## Introduction

The human being can be considered a super-organism made up of its own cells and its commensal symbiotic microbiota. These complex communities of microorganisms, which include bacteria, archaea, viruses, fungi, and other eukaryotes, not only mediate physiologically important transformations related to nutrient and drug metabolism, but also play a fundamental role in modulating the immune system and behavior (Wastyk et al., 2021). In this sense, the gut harbors a sophisticated ecosystem of microbial communities (microbiota), exerting vital metabolic functions that contribute to the recovery of nutrients and energy from indigestible substrates (Jumpertz et al., 2011; Asnicar et al., 2021). Likewise, microbial colonization is essential for the normal development of the immune system, regulating homeostasis between environmental antigenic load and immune response (Dickson, 2017). It is well studied that the immune system is trained and modulated by the commensal microbiota, having a direct impact on human health (Baruch et al., 2021). Changes in the microbiome can lead to immune dysregulation pathologies, including chronic inflammatory bowel diseases (de Souza and Fiocchi, 2016; Dickson, 2017). It is further known that the microbiome contributes to the development of the host and its physiological stability in frequent environments (Ford et al., 2020). However, it has been hypothesized that when encountering a new stressful environment, the microbiome adapts much faster than the host, disrupting its cooperation, promoting host destabilization, and generating reciprocal changes in humans and their microbiome (Soen, 2014; Suzuki and Ley, 2020). In this sense, microbiological changes induced by many perturbations are stochastic, and therefore lead to transitions from stable to unstable community states (Charlson et al., 2010; Chen et al., 2015; Zaneveld et al., 2017). According to the original canalization (stabilization) hypothesis (Waddington, 1942), after chronic prolonged exposure to the altered environment, the modified microbiome will continue to change with its host until they become sufficiently adapted to the altered environment and to one another. This hypothesis suggests that a new stability of the adapted patterns would be generated, while the variability of the microbiome would be promoted, which could be beneficial in new stressful conditions. This would allow the host to balance the stability and flexibility of their commensals based on contextual demand (Soen, 2014; Peterson et al., 2015; He et al., 2019).

In particular, lifestyle, diet, antibiotic use, and host genotype are known to condition the human microbiome (Lin, 2015), but there is a paucity of information about the microbiome of the South American populations. In this sense, most of the data related to human microbiomes were studied in European, Asian and North American populations that differ both in the genetic background and in various environmental factors with those of South America. Moreover, the heterogeneous genetic ancestry of the latter population and its rich environmental diversity account for geographical differences in the microbiota composition, as reported by several studies of Amerindian and non-Amerindian communities (Clemente et al., 2015; Santiago-Rodriguez et al., 2015; Carbonetto et al., 2016). In particular, BA and its metropolitan area constitute a megalopolis, being the second most populated urban area in the southern hemisphere after São Paulo in Brazil (United Nations Department of Economic and Social Affairs, 2019). In the absence of microbiota data in our population, our group has recently described the uncharacterized gut microbiome of BA general population (Belforte et al., 2019). Additionally, we have recently published the first local work showing the relationship between changes in intestinal microbiota and psoriasis population in Argentina (Dei-Cas et al., 2020).

It has been reported that humanity through the years has lost variability and richness in its microbiome (Smits et al., 2017), which could increase our predisposition to the development of chronic inflammatory pathologies (Ott et al., 2004; Kostic et al., 2015; Nylund et al., 2015), but also imply the irrecoverable loss of certain bacterial taxa that belonged to our microbiome in ancient times (Wibowo et al., 2021). This process would be increasingly marked in most of the world’s populations. But the most affected would be those belonging to industrialized countries, where excessive cleaning and reduced contact with nature and animals, prevents the possibility of exchange of microorganisms with biodiverse environments (Azad et al., 2013; Ege, 2017).

However, addressing multifactorial origin disorders is difficult when humanity has such diverse sociocultural customs, which represents a challenge to define international standards of health and disease. Even more so, when the world population is affected as a whole by viral infectious phenomena that are difficult to control. At the end of 2019, a novel coronavirus designated as SARS-CoV-2 emerged in the city of Wuhan, China, and caused an outbreak of unusual viral pneumonia. Being highly transmissible, this novel coronavirus disease, also known as COVID-19, has spread fast all over the world (Hu et al., 2021). COVID-19 was declared a global pandemic by the World Health Organization (WHO) on March 11th 2020, posing a great challenge to global public health. In this scenario, an association between gut microbiota composition, cytokine levels, and inflammatory markers in COVID-19 patients was recently reported, suggesting that the gut microbiome is involved in the magnitude of COVID-19 severity, possibly through modulation of host immune responses (Yeoh et al., 2021). In this sense, it has been reported that the gut microbiota could impact the antiviral immunity by affecting both the number and function of immune cells and interferon production (Sencio et al., 2021). In the lung, gut microbial composition may help control viral respiratory infections through numerous mechanisms (e.g., type I interferons production and microbial metabolites) (Luoto et al., 2014; Antunes et al., 2019; Sencio et al., 2021). Alterations in the fecal microbiota likely influence on the fecal levels of SARS-CoV-2 and the severity of COVID-19 (Zuo et al., 2020). Therefore, analysis of changes in the microbiota during SARS-CoV-2 infection can help predict patient outcomes and allow the development of microbiota-based therapies (He et al., 2020).

Concurrent with the search for effective vaccines and drug therapies, nutritional strategies (e.g., fermented foods, probiotics and prebiotics) could promote immunity (Tillisch et al., 2013; Azad et al., 2018) and are being discussed all around the globe (Calder, 2021). Understanding the variation in the microbiome as a result of behavioral changes (daily use of household disinfectants, social distancing, and dietary habit) during the pandemic, could provide new insights to understand responses to stress and perturbations and could deepen our understanding of feasible interventions for its restoration in the future.

Governments adopted quarantine measures to meet the pandemic, limiting people’s mobility and promoting individual protective behaviors such as physical distancing, the use of face masks, and hand washing (Cameron-Blake, 2021; Petherick et al., 2021). The emphasis on personal hygiene led to extraordinary daily use of detergents, disinfectants, and household cleaning products. These actions, useful to prevent the development of this infection, also affect our microbiota (Ejtahed et al., 2020). Likewise, social distancing and the loss of daily situations that allow contact between individuals is expected to affect the transfer of microorganisms within the population. Protective behaviors did not develop uniformly, but instead registered temporal variations throughout the pandemic. For example, in a global-scale study, the use of face masks registered a constant increase throughout the pandemic, while physical distancing followed an increase-decrease-increase pattern (Petherick et al., 2021). Together, these changes are expected to impact the composition and diversity of the microbiota at the individual and collective level, and will therefore have a direct impact on public health in the post-pandemic.

It should be noted that quarantines, as a context of application of those behaviors, varied in their duration and intensity (Plümper and Neumayer, 2020). In Argentina, the first COVID-19 case was confirmed in BA city on March 3rd, 2020. By March 20th, the president decreed defining rules for the PCSI, such as closing of schools and workplaces, cancelation of public events, restrictions on public and private meetings, policy of use of a face mask in public, closed and outdoor spaces, requirements to stay at homes, restrictions on internal movements, control of international travel, and prohibition of outdoor exercise. This situation remained until at least October 2020. Thus, Argentina can be included within the group of countries with the most extended measures in time, which included 119 days of strict nation-wide lock-down, 304 days of less restrictive lock-downs, and 35 days of curfews, and also the most restrictive one during 2020 (Cameron-Blake, 2021). Moreover, PCSI, which lasted for more than 7 months and was aimed at mitigating the advance of COVID-19, showed an impact on both the general health and the psycho-emotional well-being of our population (Giardino et al., 2020; Plümper and Neumayer, 2020; Etchevers et al., 2021; Llovet et al., 2021; Pisula et al., 2021). Even more, the most prolonged lockdown was accompanied by one of the largest death rates in terms of death per million inhabitants. By August 21st, 2020, in terms of total deaths, Argentina surpassed Uruguay, a neighboring country whose capital is at a similar latitude than BA and who did not mandate lock-downs or curfews, and Sweden, a country that did not use mobility restriction at all (Larrosa, 2021; Sagripanti and Aquilano, 2021). The variety and extent of these measures implemented by the government to control COVID-19 in Argentina were exceptional, making this country the best example to analyze the evolution of COVID-19 under the most stringent and longer-lasting restrictive policies. Therefore, the objective of our project was to study the gut microbiota of samples collected during PCSI in 2020, in a group of subjects from our general population previously studied during the pre-pandemic (Belforte et al., 2019), in order to know its impact both at the individual and population levels. This two-time point analysis represents an unprecedented opportunity to evaluate intra individual and population variability of the intestinal microbiota in the face of drastic changes in the environmental context, providing support for the development of possible personalized and specific nutraceuticals, functional food, probiotics or similar therapies for our population.

## Materials and Methods

### Ethics Statement

This study received approval by the Ethics Committees of Hospital Español of BA (pre-Pandemic population) and Universidad Nacional de Luján (Pandemic population), according to local regulations and Helsinki declaration. Written informed consent was obtained from all study participants.

### Epidemiological and Mobility Data Collection

The daily confirmed cases were collected from the public data of the government of the city of Buenos Aires: https://data.buenosaires.gob.ar/dataset/casos-covid-19. Mobility reports were obtained from the Buenos Aires public transportation usage database: https://data.buenosaires.gob.ar/dataset/sube.

### Study Participants

A two-time point analysis of the fecal microbiota of BA inhabitants was carried out to compare pre-pandemic data and its variation during PCSI in 2020. To this end, 23 healthy subjects, who were previously studied by our group in 2016 (Belforte et al., 2019), were recruited for a second time during the COVID-19 pandemic. Individuals that have received antibiotic therapy in the last 3 months, extreme diets (macrobiotic, vegan), history of gastrointestinal surgery (gastrectomy, bariatric surgery, colostomy), pregnancy, neoplasia, patients in therapy of renal replacement, transplanted or HIV patients were not invited to participate in the study. A COVID-19 diagnosis or a positive SARS-COV-2 test result before recruitment was not considered to be exclusion criteria for the study.

Demographic, anthropometric and socioeconomic (education level and income) characteristics as well as self-reported ethnicity were established by survey. Additionally, Goldberg Anxiety and Depression Scale was used to assess anxiety and depression in the PG. This questionnaire is based on responses of “yes” or “no” to nine depression and nine anxiety items, asking how respondents have been feeling in the past month. Goldberg et al. (1988) considered patients with anxiety scores of 4 or more or with depression scores of 2 or more as having a 50% chance of a clinically important disturbance. Higher point values indicate a more severe problem with 9 as the highest possible value for each subscale (Goldberg et al., 1988).

### Sample Collection and Microbial DNA Extraction

Participants were instructed on the collecting method of stool samples by receiving a standardized written protocol. Approximately 5 g of stool were collected in a sterile bacteriostatic buffer tube in 2016 (between 21st July and 26th August) and between days 111 (21st July, 2020) and 167 (14th September, 2020) of the PCSI (Gray et al., 2013). Specific time points of each subject’s sample collection are shown in Table 1.

**TABLE 1:** Comparison of sample collection dates, DNA extraction kits, library preparation and high-throughput sequencing methodologies for the PPG and PG analyzed in this study.

Group	Pre-pandemic (PPG)	Pandemic (PG)
Year	2016	2020
Sample collection dates		
	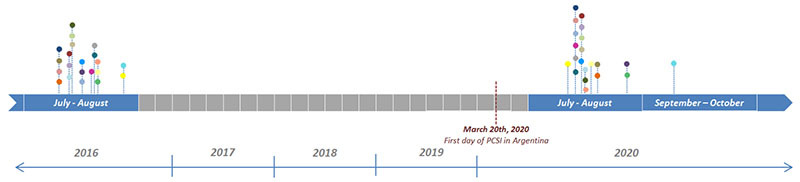	
DNA extraction kit	QIAamp DNA Stool Mini Kit (QIAGEN^®^) and Quick-DNA Soil (Zymo Research^®^)*	QIAamp-PowerFecal DNA-Kit (QIAGEN^®^)
Target region	16S gene hypervariable region V3-V4	16S gene hypervariable region V3-V4
Primers	Bakt_341F/Bakt_805R	Bakt_337F/Bakt_805R
Library preparation methodology	Herculase II Fusion DNA Polymerase Nextera XT Index Kit V2 (Illumina^®^ 16S Metagenomic Sequencing Library Preparation Part # 15044223 Rev. B)	Herculase II Fusion DNA Polymerase Nextera XT Index Kit V2 (Illumina^®^ 16S Metagenomic Sequencing Library Preparation Part # 15044223 Rev. B)
NGS chemistry (paired-end approach)	Illumina^®^ MiSeq v3 (2 × 300)	Illumina^®^ MiSeq v3 (2 × 300)
Sequences per sample (mean ± SD)	111620.15 ± 9328.76	82752.34 ± 7925.77

*All 23 stool samples were subjected to DNA extraction by both methods.

*SD, standard deviation.*

DNA samples from the “pre-pandemic group” (PPG) were obtained from 200 mg of feces using two different commercial kits for each subject [QIAamp DNA Stool Mini Kit (QIAGEN^®^) and Quick-DNA Soil (Zymo Research^®^)] following manufacturer’s instructions (Belforte et al., 2019). On the other hand, DNA samples from the “pandemic group” (PG) were obtained from 200 mg of feces using QIAamp-PowerFecal DNA-Kit (QIAGEN^®^) following manufacturer’s instructions. The concentration and purity of the nucleic acids were determined by a Qubit fluorometer (ThermoFisher^®^).

### Determination of SARS-CoV-2 Infection

Subjects were never confirmed as SARS-CoV-2 positive before recruitment and sample collection. None of the participants of the PG reported symptoms of COVID-19, were diagnosed with this disease or had a positive SARS-COV-2 test result before those time points.

At recruitment, SARS-Cov-2 infection was determined in the collected stool samples from the PG as follows. The total RNA of all stool samples collected during the PCSI was extracted by using the TRIzol reagent as previously described (Won et al., 2020). RNA was converted into complementary DNA (cDNA) using the SuperScript™ III First-Strand Synthesis System (ThermoFisher Scientific, Waltham, MA United States) following the manufacturer’s recommended procedures.

The detection of SARS-CoV-2 was carried out by partial amplification of target genes (RdRP, N, E, and S) as previously described (Park et al., 2020). A 101-base pair (bp) PCR product of the RdRP gene was amplified using primers 5′-AGAATAGAGCTCGCACCGTA-3′ (forward) and 5′-CTCCTCTAGTGGCGGCTATT-3′ (reverse). Primers for the amplification of a 117-bp PCR product of the N gene were 5′-CAATGCTGCAATCGTGCTAC-3′ (forward) and 5′-GTTGCGACTACGTGATGAGG-3′ (reverse); whereas primers for the amplification of a 116-bp PCR product of the E gene were 5′-TTCGGAAGAGACAGGTACGTTA-3′ (forward) and 5′-AGCAGTACGCACACAATCG-3′ (reverse). For the S gene, a 107-bp PCR product was amplified using primers 5′-GCTGGTGCTGCAGCTTATTA-3′ (forward) and 5′-AGGGTCAAGTGCACAGTCTA-3′ (reverse). SARS-CoV-2_IBS_E2. In these PCR protocols, 5 ng of cDNA was used as a template with the following PCR cycling conditions: 94 °C for 3 min, 35 cycles of 94 °C for 30 s, 62 °C for 40 s, and 72 °C for 1 min, with the final elongation step at 72 °C for 5 min. The component concentrations for the 50 μl final volume PCR reactions were as follows: 100 nM each primer, 0.2 mM dNTPs, 1X Colorless GoTaq Reaction Buffer which contained 1.5 mM MgCl2 (Promega, Madison, WI, United States) and 1.25U GoTaq polymerase (Promega). Finally, PCR products were visualized after electrophoresis in 2% ethidium bromide-stained agarose gels at 130 volts for 20 min.

The positive control used in the PCR reactions was pure SARS-CoV-2 viral RNA obtained from a clinical nasopharyngeal swab, which was kindly donated by the Diagnostics and Treatment Department of the Italian Hospital of Buenos Aires.

Presence of SARS-CoV-2 infection was not determined in clinical nasopharyngeal swabs at the moment of recruitment.

### Comparison of Microbial Communities and Sequence Analysis

Hypervariable regions V3–V4 of the 16S rRNA gene were amplified and sequenced in paired-end mode (2 × 300) using a MiSeq sequencer (Illumina^®^). From the 46 stool samples obtained from 23 subjects in 2016 and 2020, a total of 69 sets of sequences were analyzed: 46 sequences from 2016 (as DNA samples were obtained using two different commercial kits) and 23 from 2020. Comparison of DNA extraction kits, primers for 16S rRNA amplification, library preparation methodology, NGS chemistry and paired-end approach used in 2016 and 2020 are shown in Table 1.

Sequences generated were analyzed using Quantitative Insights Into Microbial Ecology (QIIME2) version 2021.2 software package (Bolyen et al., 2019). Raw fastq reads were quality filtered (denoised, merged, and assessed for chimeras) to produce amplicon sequence variants (ASV) using the DADA2 (Plugin version 2021.2.0) pipeline (Callahan et al., 2016). Figaro software was used to determine optimal trimming parameters for each group (trunc-len for PPG and PG samples was f271 r213 and f265 r219, respectively) (Weinstein et al., 2020). After rare amplicon sequence variant filtering [0.1% minimum abundance filter was chosen based on the known 0.1% bleed through between Illumina MiSeq runs (Laurence et al., 2014; Salter et al., 2014)], tables were merged. The within-sample (alpha) diversity was determined using the QIIME2 q2-diversity plugin. Alpha and beta diversity were calculated using genus-level data in a single rarefaction to the sample with the lowest sequence depth at 29,753 sequences. In order to place each sequence into a reference phylogenetic tree, qiime fragment-insertion SEPP (version 4.3.10) was used (sepp-refs-silva-128.qza reference database) (Mirarab et al., 2012). To perform the taxonomic classification by qiime feature-classifier classify-sklearn, we train a supervised learning classifier with RESCRIPt package (Janssen et al., 2018; Robeson et al., 2020), using the V3-V4 primers from this study and a 99% similarity threshold following the author’s tutorial^1^. The database used for this taxonomic assignment was Silva Release 138 (Quast et al., 2013). Alpha and beta diversity were calculated using qiime diversity core-metrics-phylogenetic pipeline. Differences on beta diversity were assessed using ADONIS permutation-based statistical test in vegan-R^2^ implemented in QIIME2 (q2-diversity plugin 2021.2.0) (Anderson, 2001). A standard pipeline of Phylogenetic Investigation of Communities by Reconstruction of Unobserved States (PICRUSt version 2.4.1), implemented in QIIME2, was used to generate MetaCyc pathway ontology profiles (Caspi et al., 2016; Douglas et al., 2020). Diversity core-metrics were calculated using predicted MetaCyc data rarefied to the sample with the lowest count (2594868 features). Differences in taxa abundance at the phylum level and functional profiles between PPG vs. PG were determined using the analysis of composition of microbiomes (ANCOM) framework (Mandal et al., 2015). Core microbiota was defined as the set of amplicon sequence variants detected in 50--100% of the samples with a relative abundance threshold value above 0.01% (calculated with Core microbiome from R microbiome package). Data are presented either as direct visualization of QIIME2 artifacts on QIIME2 View, or using ggplot2 (version 3.3.1) with data extracted from QIIME2 artifacts by using qiime2R (v0.99.5).^3^ Posterior analysis was realized with phyloseq [version 1.34.0, (McMurdie and Holmes, 2013)] and microbiome [version 1.12.0, (Lahti et al., 2017)] R packages. The complete pipeline including all parameters used for data analysis is described in detail in Supplementary File 1.

### Data Accession

Raw sequences of 16S rRNA gene reported in this article have been deposited in NCBI Short Read Archive (SRA) and are accessible under PRJNA503303 (Samples C101Q, C101Z, C128Q, C128Z, C129Q, C129Z, C131Q, C131Z, C132Q, C132Z, C133Q, C133Z, C134Q, C134Z, C135Q, C135Z, C136Q, C136Z, C137Q, C137Z, C138Q, C138Z, C140Q, C140Z, C141Q, C141Z, C142Q, C142Z, C144Q, C144Z, C145Q, C145Z, C147Q, C147Z, C150Q, C150Z, C151Q, C151Z, C153Q, C153Z, C155Q, C155Z, C156Q, C156Z, C157Q and C157Z) and PRJNA763205 (Samples ASPO-C101, ASPO-C128, ASPO-C129, ASPO-C131, ASPO-C132, ASPO-C133, ASPO-C134, ASPO-C135, ASPO-C136, ASPO-C137, ASPO-C138, ASPO-C140, ASPO-C141, ASPO-C142, ASPO-C144, ASPO-C145, ASPO-C147, ASPO-C150, ASPO-C151, ASPO-C153, ASPO-C155, ASPO-C156, ASPO-C157) accession numbers for PPG (year 2016) and PG (year 2020), respectively.

### Statistical Analysis

Data for continuous variables are shown as means ± SD. Categorical variables are reported as proportions (%). Statistical analyses were performed using R (version 4.0.5). Differences between groups for categorical and continuous metadata were evaluated using Fisher’s exact test and Mann–Whitney *U*-tests, respectively.

## Results

### COVID-19 Pandemic in Buenos Aires and Background of Study Cohort

All subjects were Argentines of European descent, residents of BA city and its metropolitan area, the second most populated agglomeration in the southern hemisphere. Analysis of their socioeconomic background revealed that 91% of the 23 recruited subjects held a university degree and had the highest income level, according to the World Bank Data for Argentina^4^.

Of the 23 recruited subjects, 11 of them were men (47.8%). All subjects had omnivorous diets.

Subjects originally studied in July–August 2016 were re-recruited during the months of July through September 2020 (Table 1), when COVID-19 cases peaked in BA city despite the restricted measures implemented since the month of March (Supplementary Figure 1).

The anthropometric characteristics of the subjects at the two-time points analyzed in the study are shown in Table 2. No statistically significant differences were observed when the age, body mass index and its categories were compared between 2016 and during PCSI in 2020 (*p* = 0.55) (Table 2).

**TABLE 2:** Anthropometric characteristics of the subjects at the two-time points analyzed in the study.

Characteristics	PPG (2016)	PG (2020)	*p*-value
Age, years, mean ± SD	35.87 ± 8.87	39.87 ± 8.87	0.13
BMI, kg/m^2^, mean ± SD	23.29 ± 2.82	23.84 ± 3.07	0.18
Normal weight (<25 kg/m^2^), *N*,%	15, 65.2%	12, 52.2%	0.55
Overweight (25–29.9 kg/m^2^), *N*,%	8, 34.8%	11, 47.8%	

*SD, standard deviation; BMI, body mass index.*

At the moment of subjects’ re-recruitment in 2020 (130.47 ± 12.46 days of PCSI), 56.5% of volunteers (13 out of 23) reported probable anxiety, whereas 5 of them (5 out of 13; 38.5%) recorded 7 or 8 points indicating a more severe problem, according to the Goldberg Anxiety and Depression Survey. Regarding the depression items of the same questionnaire, 60.9% of the subjects (14 out of 23) reported probable depression, with 2 of them (8.7%) scoring 6 and 7 out of 9 points.

The presence of SARS-CoV-2 was not detected in any stool sample. PCRs targeting SARS-CoV-2 RdRP, N, E, and S genes rendered negative results in all samples. PCR adequate execution and the accuracy of the obtained results were also controlled and confirmed by positive and negative controls, which always exhibit appropriate results.

### Sequence Analysis and Comparison of Microbial Communities

The hypervariable region V3-V4 of bacterial 16S gene was sequenced using MiSeq-Illumina system, obtaining 111620.15 ± 9328.76 and 82752.34 ± 7925.77 sequences per sample for PPG and PG, respectively.

**FIGURE 1:**
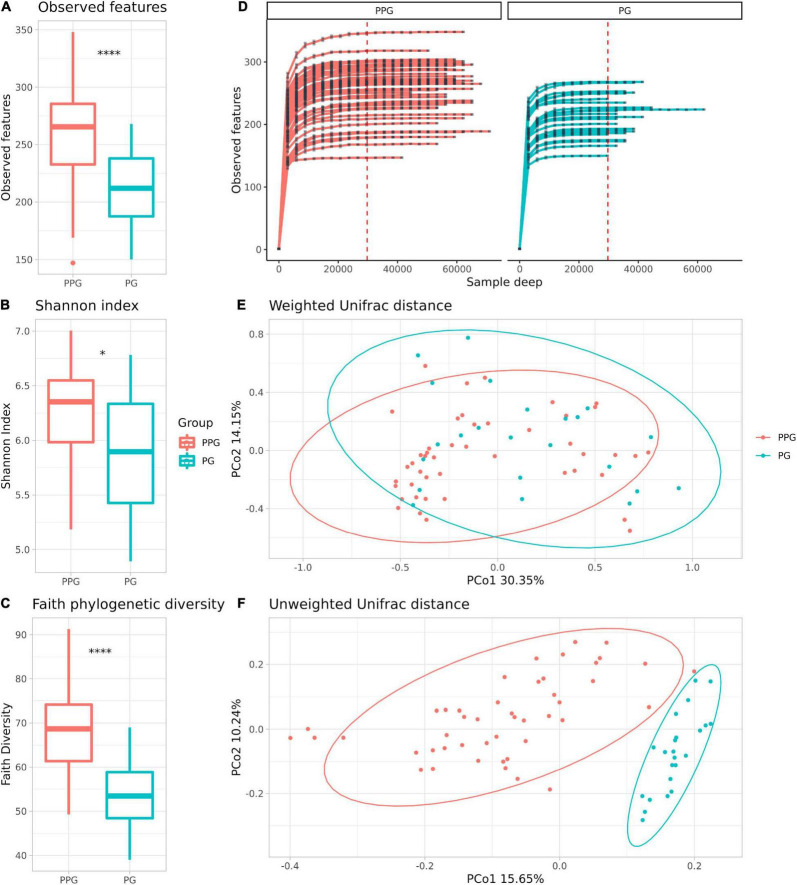
Comparison of the microbiome community of PPG and PG groups. Alpha Diversity measures: Observed features **(A)**, Shannon index on a base-2 logarithmic scale **(B)** and Faith phylogenetic diversity **(C)**. *p = 0.026; ^*⁣*⁣**^p < 0.001. Rarefaction curves of the samples from the PPG and PG **(D)**. The x axis represents the number of sequences sampled while the y axis represents a measure of the species richness detected (estimated number of observed features). The red vertical dotted line represents the rarefaction depth chosen (sample with the least amount of sequences). PCoA plots of beta diversity with weighted **(E)** and unweighted **(F)** UniFrac distances, respectively. Ellipses represent the 95% confidence interval of each group. Colors are assigned by group, red for PPG and blue for PG.

Alpha-diversity is a measure of microbial richness, or the number of distinctive taxa (richness), and the distribution of the taxa, referred to as the evenness, within samples. Regarding the metrics commonly used to measure alpha diversity, observed features measures richness, that is the number of different taxa you observe in a sample at a given taxonomic level, Shannon entropy index measures richness and evenness of samples, and Faith’s PD quantitatively measures richness using phylogenetic relationships within the samples (Willis, 2019). In this study, we found significant differences in the estimated number of observed features (*p* < 0.001; Figure 1A), Shannon entropy index (*p* = 0.026; Figure 1B) and in Faith phylogenetic diversity (Faith’s PD) (*p* < 0.001; Figure 1C) between PPG vs. PG, being significantly lower in the PG. Rarefaction plots reached an asymptotic state, indicating that the sequence depth was sufficient to represent the bacterial community richness and diversity (Figure 1D).

Beta diversity (considering weighted and unweighted UniFrac distances) was analyzed in order to compare the differences between microbial compositions among different groups (Figures 1E,F and Supplementary Figure 2). The PPG and PG show a clear separation only in the PCoA unweighted Unifrac plot (Figure 1E), which is a qualitative measure which uses only the presence/absence of data to estimate the distance between two communities showing when communities differ primarily by which microorganism can live in them, being thus capable to detect effects of restrictive factors for microbial growth. In contrast, weighted UniFrac is a quantitative measure that accounts for the relative abundance of microbial lineages and can reveal the effects of more transient factors (Lozupone et al., 2007). Differences on beta diversity values between Groups (PPG-PG), purification kit employed, and Subject ID were evaluated (ADONIS). As shown in Table 3, Groups and Subject ID were statistically significant whereas the purification kit used for DNA extraction was considered to be not significant in the unweighted UniFrac. On the other hand, in the weighted UniFrac all comparisons were statistically significant. However, the *R*^2^-value indicates that in both metrics, the most important effect on the variation was the Subject ID, followed by the Group pertinence. The purification kit used for DNA extraction had no significant effect on the unweighted UniFrac and marginal effects on the weighted UniFrac (Table 3).

**TABLE 3:** Results of permutational multivariate analysis of variance (Adonis) using weighted and unweighted UniFrac dissimilarity matrices using beta diversity values between Groups (PPG-PG), purification kit for DNA extraction employed, and Subject ID.

	Unweighted UniFrac						Weighted UniFrac					
	*df*	Sum Sq.	Mean Sq.	F-model	*R* ^2^	Pr(> F)	df	Sum Sq.	Mean Sq.	F-model	*R* ^2^	Pr(> F)
Groups	1	1.3	1.3	17.1	0.1	**0.001**	1	1.6	1.6	5.7	0.04	**0.001**
Purification kit	1	0.1	0.1	0.9	0.006	0.5	1	0.6	0.6	2.1	0.01	0.04
Subject ID	22	6.4	0.3	3.8	0.6	**0.001**	22	24.5	1.1	4.1	0.6	**0.001**
Residuals	43	3.3	0.1		0.3		43	12.13	0.3		0.3	
Total	67	11.2			1		67	38.8			1	

*Degrees of freedom (df) corresponds to one less than the number of values in the set of means. The p-values are derived from the F distribution and the significant level Pr(> F) < 0.05 are presented in bold.*

In PPG, the dominant phyla were Bacteroidota (44.14 ± 8.72%), Firmicutes (41.87 ± 9.36%), Proteobacteria (6.63 ± 4.57%), Verrucomicrobia (2.26 ± 1.93%) and Actinobacteria (2.26 ± 1.93%), whereas the principal phyla found in PG were Firmicutes (47.64 ± 11.72%), and Bacteroidota (40.32 ± 13.45%), followed by Proteobacteria (5.18 ± 7.57%), Actinobacteria (5.32 ± 7.38%) and Verrucomicrobia (0.92 ± 1.30%) (Supplementary Figure 3). When the main detected phyla (ANCOM) were compared between groups, a significant decrease in phylum verrucomicrobia was observed during the PCSI. In this sense the F/B ratio was also altered, being 1.01 ± 0.38 for PPG and 1.47 ± 0.96 for PG (Mann Whitney *U*-test, *p* = 0.042).

### Impact of Preventive and Compulsory Social Isolation in Core Microbiota and Differential Abundant Taxa Between Pre-pandemic Group and Pandemic Group

**FIGURE 2:**
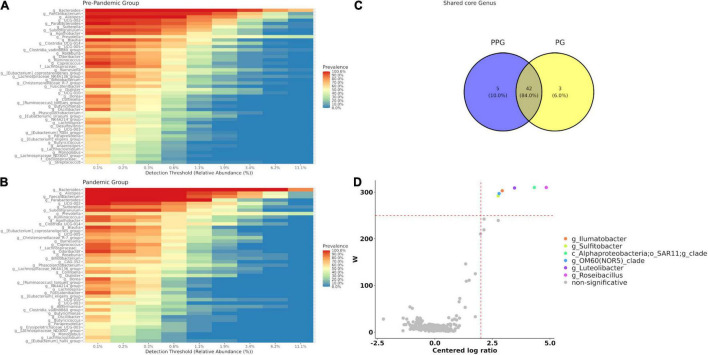
Core microbiome for each group PPG **(A)** and PG **(B)**. **(C)** Venn diagram represents shared care genera between groups. Core genera were defined as 0.1% of detection and 50% of prevalence. **(D)** Volcano plot of the differentially abundant genera between PPG and PG patients. The *W*-value represents the number of times the null-hypothesis (the average abundance of a given feature in a group is equal to that in the other group) was rejected for a given feature. Red dash lines indicate a significance *a priori* threshold for differentially abundance set at *W* ≥ 248 (*W* > 80% of the total number of genera) and clr (centered logarithmic ratio) > | 2|. Significant genera more abundant in PPG are represented in different colors; whereas non-significant genus are in gray.

Forty-seven bacterial genera (corresponding to 16.96% of the genera present in the group) were identified as core microbiota for the PPG (Figure 2A) and forty-five for the PG, being 16.24% of the total (Figure 2B), considering prevalence ≥ 50% and a detection threshold ≥ 0.1% (or else a frequency of ≥ 0.001). At the intersection of both groups (Figure 2C), forty-two genera were found, observing only a few core features exclusively represented in each of the groups. In this sense, there were five genera present in PPG that were lost in PG core microbiota (g__*Desulfovibrio*, g__*Streptococcus*, g__*Anaerostipes*, f__*Oscillospiraceae*;__unclasifed, g__[*Eubac* *terium*]_siraeum_group) and three genera (g__*Erysipelotr ichaceae*_UCG-003, g__CAG-352, g__*Akkermansia*) exclusively present in PG core that were not previously observed in PPG (Figures 2A–C).

The differential abundant taxa between PPG and PG using the Analysis of Composition of Microbiomes method (ANCOM) were additionally explored. As shown in Figure 2D, of the 312 observed taxa at the genus level, abundance of Roseibacillus (*W* = 310), Alphaproteobacteria SAR11 Clade_Ia (*W* = 310), Luteolibacter (*W* = 309) Ilumatobacter (*W* = 303), OM60(NOR5)_clade (*W* = 297), and Sulfitobacter (*W* = 292) showed a significant difference (*p* < 0.05) between the PPG and PG, being absent in the latter group. Finally, considering that the first linear coordinate (PCoA1) of unweighted UniFrac was able to differentiate both groups, we study the contribution of each genus and other variables from metadata. To this end, a vector correlation by fitting metadata and genera vectors on ordination space for bacterial communities was done. None of the metadata studied correlated with this axis, but the six genera that ANCOM showed as significantly decreased during the pandemic were significantly correlated with PCoA1 (*p* < 0.001; Supplementary Figure 4).

### Functional Analysis

**FIGURE 3:**
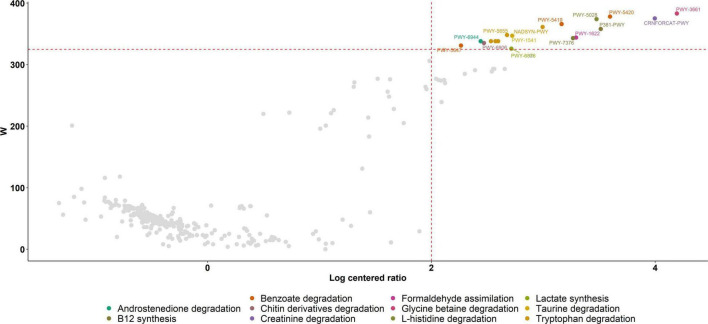
Volcano Plot of PICRUSt analysis. Significant metabolic pathways down-regulated in the gut microbiota of individuals during the PCSI are represented in different colors with their corresponding MetaCyc ID. Metabolic pathways without a statistically significant difference between groups are shown in gray. The *W*-value represents the number of times the null-hypothesis (the average abundance of a given feature in a group is equal to that in the other group) was rejected for a given metabolic pathway. Red dash lines indicate a significance *a priori* threshold for differentially abundance set at *W* ≥ 324 (*W* > 80% of the total number of metabolic pathways) and clr (centered logarithmic ratio) > |2|.

Putative functional genes were predicted using PICRUSt which applies 16S rRNA gene data to predict the abundance of functional pathways. We first explored the relationship between functional alpha diversity in PPG and PG. When we compare alpha diversity metrics in the results from functional predictions, we found a significant decrease in PPG diversity (Shannon *p* = 0.01, evenness *p* = 9.1e-08) and observed_features (*p* = 12 2.9e-10). In this sense, several genes related to metabolic pathways were less prevalent in the gut microbiota of individuals during the PCSI and these differences were statistically significant. Gut degradation of tryptophan (PWY-5655, PWY-5654, PWY-6505, NADSYN-PWY and PWY-5651), benzoate (PWY-5420, PWY-5419 and PWY-5647), glycine-betaine (PWY-3661), taurine (PWY-1541), creatinine (CRNFORCAT-PWY), L-histidine (PWY-5028), lactate (PWY-6876), chitin derivatives (PWY-6906) and androstenedione (PWY-6944) were reduced during PCSI, as well as, formaldehyde assimilation (PWY-1622) related pathways and vitamin B12 (P381-PWY, and PWY-7376) synthesis (Figure 3).

## Discussion

It is important to understand the composition and diversity of the intestinal microbiota in different contexts in order to describe it, interpret its behavior, and define potential environmental factors that affect it. In this sense, the COVID-19 pandemic poses a great challenge to global public health. The emphasis on personal hygiene led to an extraordinary daily use of detergents, household disinfectants and cleaning products (Altunisik Toplu et al., 2020). Likewise, social distancing, quarantines and lock-downs cause the loss of everyday situations that allow contact between individuals (Jefferson et al., 2020; Talic et al., 2021).

Together, these measures, that have a direct effect on the transfer of microorganisms within a given population, impact the composition and diversity of the gut microbiota in other populations of the world (Peng et al., 2021; Rashidi et al., 2021), with no data from South American populations reported at the moment. Additionally, it should be noted that these restrictive measures, as a context of application of those behaviors, varied in their duration and intensity (Plümper and Neumayer, 2020), being Argentina an exceptional example to analyze the evolution of COVID-19 under the most stringent and longer-lasting restrictive policies registered around the world (Cameron-Blake, 2021; Larrosa, 2021; Sagripanti and Aquilano, 2021). In this study, a two-time point analysis of the fecal microbiota of BA inhabitants before and during the PCSI was carried out, both at the individual and population level.

To this end, during PCSI in 2020, we re-recruited 23 healthy subjects previously studied in 2016 (Belforte et al., 2019), residents of the same urban geographical area and belonging to the same ethnicity and socioeconomical status. Aside from the expected differences on age, no statistically significant differences were observed when body mass index and its categories were compared between 2016 and during PCSI in 2020. This result is in agreement with a recent study that described diet and lifestyle changes during the COVID-19 pandemic in ibero-american countries, as Argentina showed one of the highest proportion of changes toward a healthier pattern of food consumption during the pandemic situation (Enriquez-Martinez et al., 2021), despite the fact that it has been reported that quarantine can further aggravate health status by affecting lifestyle choices including lack of physical activity and weight gain (Bhutani and Cooper, 2020).

In the PG, the emotional consequences of the isolation precautions during the pandemic have been documented by the Goldberg Anxiety and Depression Scale. Although we cannot exclude the possibility of previous undiagnosed anxiety and/or depression conditions among the subjects, these results are in agreement with several worldwide studies (Loades et al., 2020; Wu B., 2020). A systematic review of experiences of social isolation prior to COVID-19 concludes that these can have lasting consequences for people, manifested, for example, in avoidance behaviors (Brooks et al., 2020; Cameron-Blake, 2021). In another study, carried out in the elderly population of the city of Buenos Aires, referring to the impact of isolation, a greater negative emotional impact (anguish, sadness, depression, fear, loneliness) has also been documented (Llovet et al., 2021).

Although the analysis of gut microbiota among COVID-19 infected and uninfected was not the aim of this study, SARS-CoV-2 was not detected in the stool samples from the PG. However, SARS-CoV-2 infection of the PG cannot be ruled out because presence of SARS-CoV-2 infection was not determined by Real Time PCR in clinical nasopharyngeal swabs (the gold standard method of diagnosis) at the moment of recruitment. Moreover, as SARS-CoV-2 is a highly variable virus and variants continue to emerge, negative PCR results could be due to the low specificity of the toward certain viral variants (Jain et al., 2021). However, we consider this to be an unlikely situation because variants emergence was reported after sample recruitment (Aleem et al., 2022) and PCR protocols targeting 4 different genomic regions, including the highly conserved N and E genes (Mohammad et al., 2021), rendered negative results in all samples.

In this study, the structure of the microbiome community was analyzed by different alpha diversity metrics with respect to its richness and evenness. We find that samples from the PPG had higher richness and evenness than those from the PG. The trend is observed not only with phylogenetic (Faith’s phylogenetic diversity) and non-phylogenetic (observed features) richness metrics, but also in the Shannon index scores that show both the abundance and evenness of the taxa present (Figures 1A–C). The magnitude of the significant difference observed between phylogenetic and non-phylogenetic richness indices indicates that the gut microbiomes of PPG are composed of larger number of phylogenetically diverse taxa, while the gut microbiomes of subjects during PCSI are composed of fewer closely related taxa. Furthermore, these differences in richness between groups are robust to rarefaction, being detected with the lowest sequence depth.

As expected, decreased person-to-person and environment-to-person transmission of microorganisms can result not only in their lower diversity but also in a reduced abundance of specific taxa in the gut microbiota (Peng et al., 2021; Rashidi et al., 2021). In fact, although no strong change was observed in the core microbiota between the groups in this study, a significant decrease was observed during PCSI in the phylum Verrucomicrobia, a mucin-degrading bacteria residing in the intestinal mucosa that contribute to intestinal health and glucose homeostasis, and plays as an interface between the human gut microbiome and host tissues (Anderson et al., 2017). Moreover, the F/B ratio was increased during the PCSI. However, it is known that the relative abundance of the Firmicutes and Bacteroidota phyla is highly variable between subjects from the same population and it is currently difficult to associate them with a determined health status, this increased ratio is frequently associated with dysbiosis and reported among obese patients (Magne et al., 2020).

Microbial community structure (beta diversity) was also compared between PPG and PG. The differences observed in the microbiota structure by unweighted UniFrac analysis, a type of qualitative measure that can better detect detrimental factors for microbial homeostasis (Lozupone et al., 2007), could be explained by six differential abundant genera that were absent during PCSI (Figure 2D and Supplementary Figure 4).

In non-human primates, social interactions are a major determinant of the gut microbiome (Moeller et al., 2016). In human societies alike, isolation and socialization in smaller groups (e.g., within rather than among families) reduce social contacts, resulting in microbiomes that resemble those of close family members or friends (Brito et al., 2019). The increased use of disinfectants, sanitizers, and antibiotics for containment of the virus has been proposed to cause collateral damage to the gut microbiota, compromising colonization resistance and promoting the growth of antibiotic-resistant species and pathogens (Singh, 2020). The “disappearing microbiota hypothesis” predicts that reduced acquisition of microbes due to decreased exposure to the external environment (versatile foods, people, and environment) and increased use of antimicrobial agents will cause microbial diversity loss (Blaser and Falkow, 2009).

The distinctive taxa found could be responsible for the down-regulation of predicted metabolic pathways observed as a consequence of PCSI. However, it should be taken into consideration that PICRUSt’s predictive approach carried out in this study neither precludes nor outperforms deep metagenomic sequencing. It can predict and compare probable functions across many samples from a wide range of habitats at a small fraction of the cost of such sequencing (Langille et al., 2013). Therefore, the limitations of this approach must be considered when interpreting the following results of the PICRUSt predictions.

In this study, tryptophan degradation was predicted to be reduced during PCSI. In this sense, chronic stress in combination with alteration of serotonin, a regulator of several behavioral, mood and neuroendocrine functions, are elements expected to be found during PCSI, and they may accelerate the breakdown of its precursor tryptophan by human metabolism, leading to its decreased concentrations in the intestine and supporting the predicted downregulation of its degradation by the gut microbiota (Agus et al., 2018; Gostner et al., 2020).

Moreover, queuosine synthesis was predicted to be reduced during PCSI. Queuosine and its derivatives occur exclusively at position 34 (the wobble position) in the anticodons of tRNAs coding for the amino acids L-histidine, L-aspartate, L-asparagine and L-tyrosine. In fact, histidine reduction may produce feelings of fatigue, lower the performance during working memory tasks, and deteriorate clear thinking and attentiveness (Sasahara et al., 2015).

Metabolic pathways related to sodium benzoate degradation products were reduced in the PG in this study. Sodium benzoate is commonly used as a preservative in packaged food (Yadav et al., 2021). Diminished expression of benzoate degradation pathways would be related to the decrease in the consumption of foods with preservatives, following the increased consumption of homemade food during PCSI.

Additionally, reduction in both frequency and intensity of physical exercise stands out among the life-style changes forced by PCSI. The relationship of the composition of gut microbiota and physical exercise has been previously proposed. First, lactate and creatinine produced in the muscle during physical activity enters the intestinal lumen *via* the blood circulation. In the intestine, it acts as a carbon source for specific microbes, which causes the production of SCFA byproducts (predominantly propionate), which are taken up by the host *via* the intestinal epithelium. The presence of microbiome-sourced SCFAs in the blood improves athletic performance *via* an unknown mechanism (Scheiman et al., 2019). In this study, the analysis of the metabolic pathways of the gut microbiome revealed that degradation of creatinine and lactate could probably be diminished, which is in agreement with an increase in sedentarism during the PCSI.

Limitations of the present study should be considered when interpreting the results. First, we cannot rule out a possible relationship between modification of the gut microbiota composition and diversity and changes in the dietary habits when comparing PPG and PG, as diet was not recorded at both time points. Although diet is one of the most important factors that modified gut microbiota (Zmora et al., 2019), all samples were collected in the same season (winter) in 2016 and 2020 and thus, seasonal differences in the diet composition (Koliada et al., 2020) could be ruled out. Second, after dismissing bias due to different DNA extraction kits in the PPG and PG, it must be taken into consideration that the observed changes in the gut microbiota composition and diversity could be attributed to different sequencing runs (McLaren et al., 2019). Although rarefaction curves were examined and alpha and beta diversity were calculated using genus-level data in a single rarefaction to the sample with the lowest sequence depth, no sequencing controls were included to adjust differences between runs. Finally, shotgun sequencing would have provided more reliable results and species-level resolution than 16S gene metagenomics. However, the excessive cost of biotechnology renewal (sequencing platforms upgrade) for the majority of Latin American economies, results in a poor sustainability of genomic projects (Alvarez-Gomez et al., 2021) and exacerbates the paucity of shotgun metagenomic studies in our region.

In conclusion, the extensive use of antiseptic/disinfectant products along with social distancing during PCSI was expected to directly impact the microbiome, given the reduction in exposure to non-pathogenic commensal bacteria thus triggering intestinal dysbiosis. It is striking, since the second world has increased various complex pathologies with an inflammatory component such as metabolic syndrome, obesity, diabetes, inflammatory bowel diseases, allergies and even diseases with cognitive impairment such as autism (Bommer et al., 2017). Industrialization is essentially correlated with the reduction of the diversity of the human microbiota (Smits et al., 2017), which implies the loss of our ancestral microbial heritage to which we were exposed through evolution (Bello et al., 2018). The loss of microbiota diversity opens niches for opportunistic invaders, which often do not have the same coevolved constraints. Although the human microbiome has resilient properties, the response to a new disturbance of the human microbial ecosystem could negatively impact modern diseases. This context, together with the inevitable consequences of the pandemic due to the interruption of treatments in patients with pre-existing pathologies, or delay in new diagnoses resulting from the collapse of the health system, could have a synergistic effect increasing the risk of complex pathologies in the post-pandemic. Although this work has a limited sample size and is circumscribed to a specific geographical area, this data supports the hypothesis that the microbiome of the inhabitants of the metropolitan area of BA changed in the context of isolation during PCSI. This was not only observed at the diversity and taxa distribution level, but also several gut metabolic pathways were predicted to be downregulated during PCSI with potential direct consequences in human health. Moreover, these results could contribute to deepening the knowledge of the gut microbiota in order to be able to establish future interventions that allow restoring a healthy microbiota.

## Data Availability Statement

The datasets presented in this study can be found in online repositories. The names of the repository/repositories and accession number(s) can be found below: https://www.ncbi.nlm.nih.gov/bioproject/PRJNA763205; https://www.ncbi.nlm.nih.gov/bioproject/PRJNA503303.

## Ethics Statement

This study received approval from the Ethics Committees of Hospital Español of BA (pre-pandemic population) and Universidad Nacional de Luján (Pandemic population), according to local regulations and Helsinki declaration. Written informed consent was obtained from all study participants.

## Author Contributions

FB, JT, and AP-S designed the study. FB and AP-S performed the recruitment of the volunteers. AR, MM, and SQ collected stool samples and performed the extraction of fecal bacterial DNA. GI carried out the high-throughput sequencing. PA, AR, and AP-S processed the raw sequences and performed the bioinformatic and statistical analysis. FB, MM, IL, JT, and AP-S analyzed the results and wrote the manuscript. All authors contributed to the article and approved the submitted version.

## Conflict of Interest

The authors declare that the research was conducted in the absence of any commercial or financial relationships that could be construed as a potential conflict of interest.

## Publisher’s Note

All claims expressed in this article are solely those of the authors and do not necessarily represent those of their affiliated organizations, or those of the publisher, the editors and the reviewers. Any product that may be evaluated in this article, or claim that may be made by its manufacturer, is not guaranteed or endorsed by the publisher.

## Footnotes

1 Bokulich-lab. (2022). *GitHub - Bokulich-lab/RESCRIPt: REference Sequence Annotation and CuRatIon Pipeline*. San Francisco, CA: GitHub.

2
https://CRAN.R-project.org/package=vegan

3
https://github.com/jbisanz/qiime2R/

4 Argentina (2022). *Argentina*. Available Online at: https://data.worldbank.org/country/AR (accessed January 31, 2022).

## Supplementary Material

Supplementary Figure 1 Number of COVID-19 cases, confirmed deaths and public transportation usage in Buenos Aires city in 2020. The pink bar represents the first day of PCSI in Argentina.

Supplementary Figure 2 PCoA plots of beta diversity, **(A)** weighted PCoA1 vs. PCoA3 **(B)** weighted PCoA2 vs. PCoA3 UniFrac distances and **(C)** unweighted PCoA1 vs. PCoA3 **(D)** unweighted PCoA2 vs. PCoA3 UniFrac distances, respectively. Ellipses represent the 95% confidence interval of each group. Colors are assigned by group, red for PPG and blue for PG.

Supplementary Figure 3 Relative abundance and prevalence of the different phyla of the gut microbiota in each subject in the PPG and PG.

Supplementary Figure 4 Unweighted UniFrac PCoA plots of beta diversity with significant vector correlation by fitting metadata and ASVs vectors on ordination space for bacterial communities. Factors shown had a significant correlation with ordination axes (*p* < 0.05).

Supplementary File 1 Pipeline for data analysis used in this study.
