# Supplementary figures and images for: A Two-Time Point Analysis of Gut Microbiota in the General Population of Buenos Aires and Its Variation Due to Preventive and Compulsory Social Isolation During the COVID-19 Pandemic
Source: Front Microbiol. 2022 Mar 24;13:803121. doi: 10.3389/fmicb.2022.803121 (PMC8988235; doi:10.3389/fmicb.2022.803121)

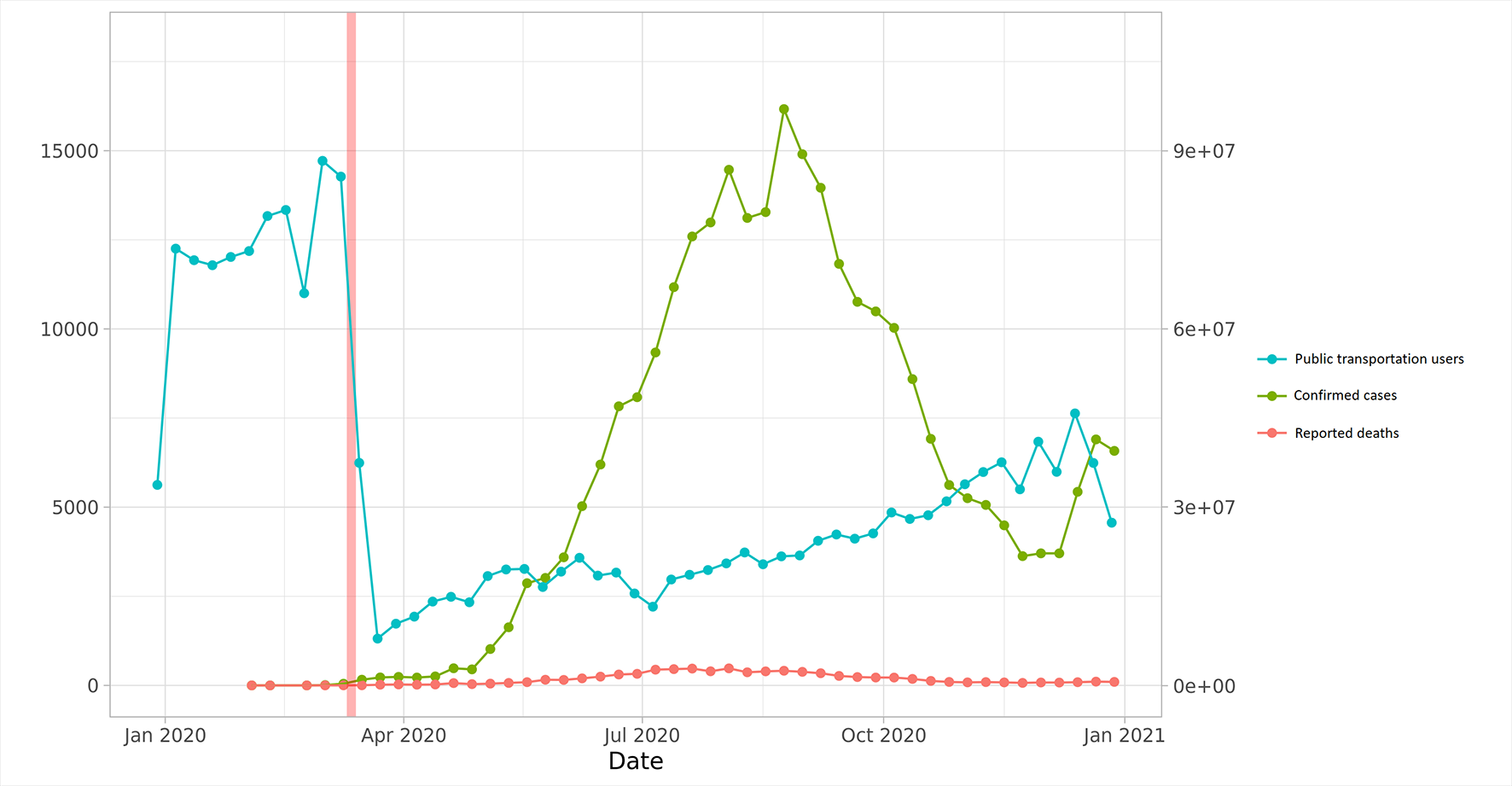

Supplement: Supplementary Figure 1 — Number of COVID-19 cases, confirmed deaths and public transportation usage in Buenos Aires city in 2020. The pink bar represents the first day of PCSI in Argentina. [file Image_1.TIF]

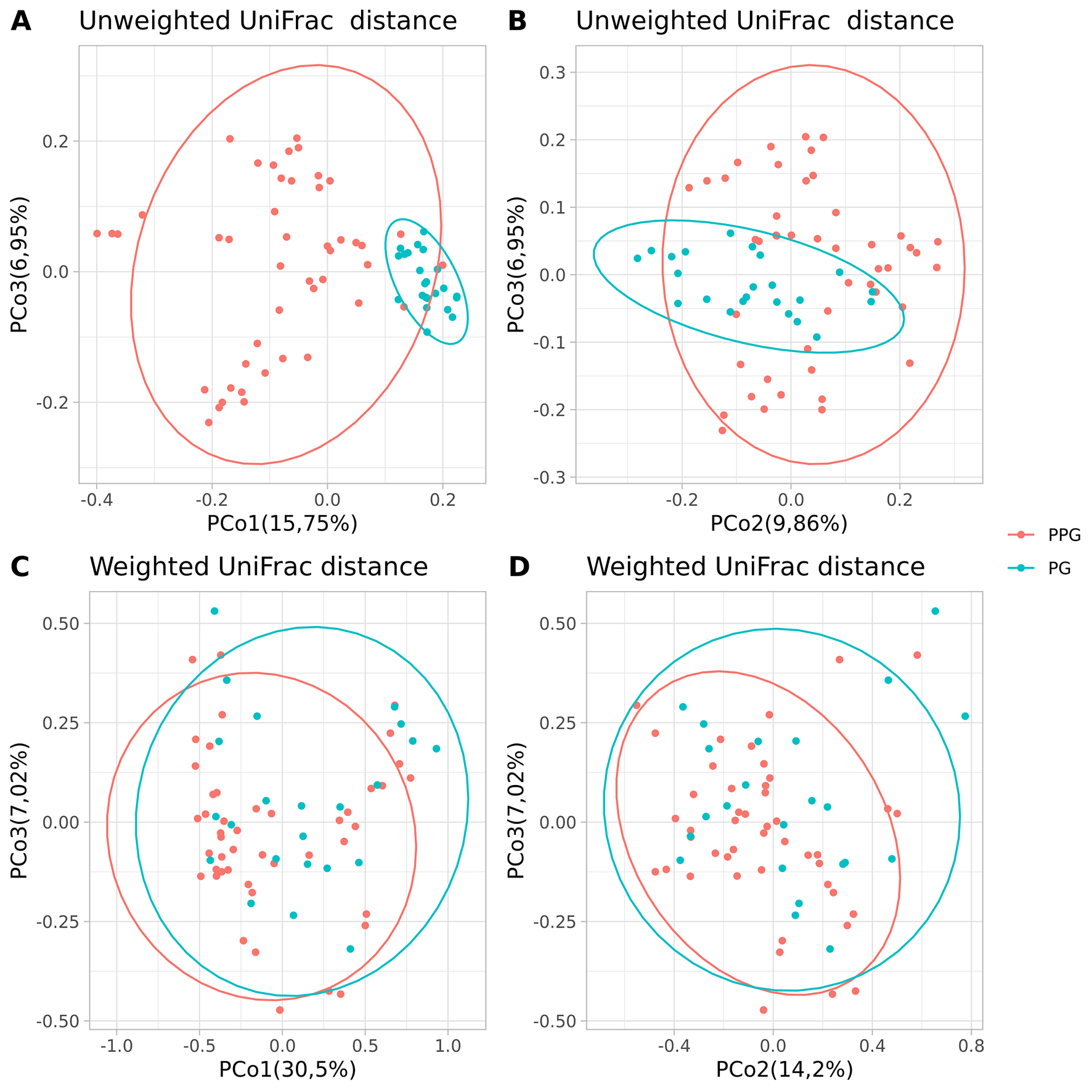

Supplement: Supplementary Figure 2 — PCoA plots of beta diversity, (A) weighted PCoA1 vs. PCoA3 (B) weighted PCoA2 vs. PCoA3 UniFrac distances and (C) unweighted PCoA1 vs. PCoA3 (D) unweighted PCoA2 vs. PCoA3 UniFrac distances, respectively. Ellipses represent the 95% confidence interval of each group. Colors are assigned by group, red for PPG and blue for PG. [file Image_2.TIFF]

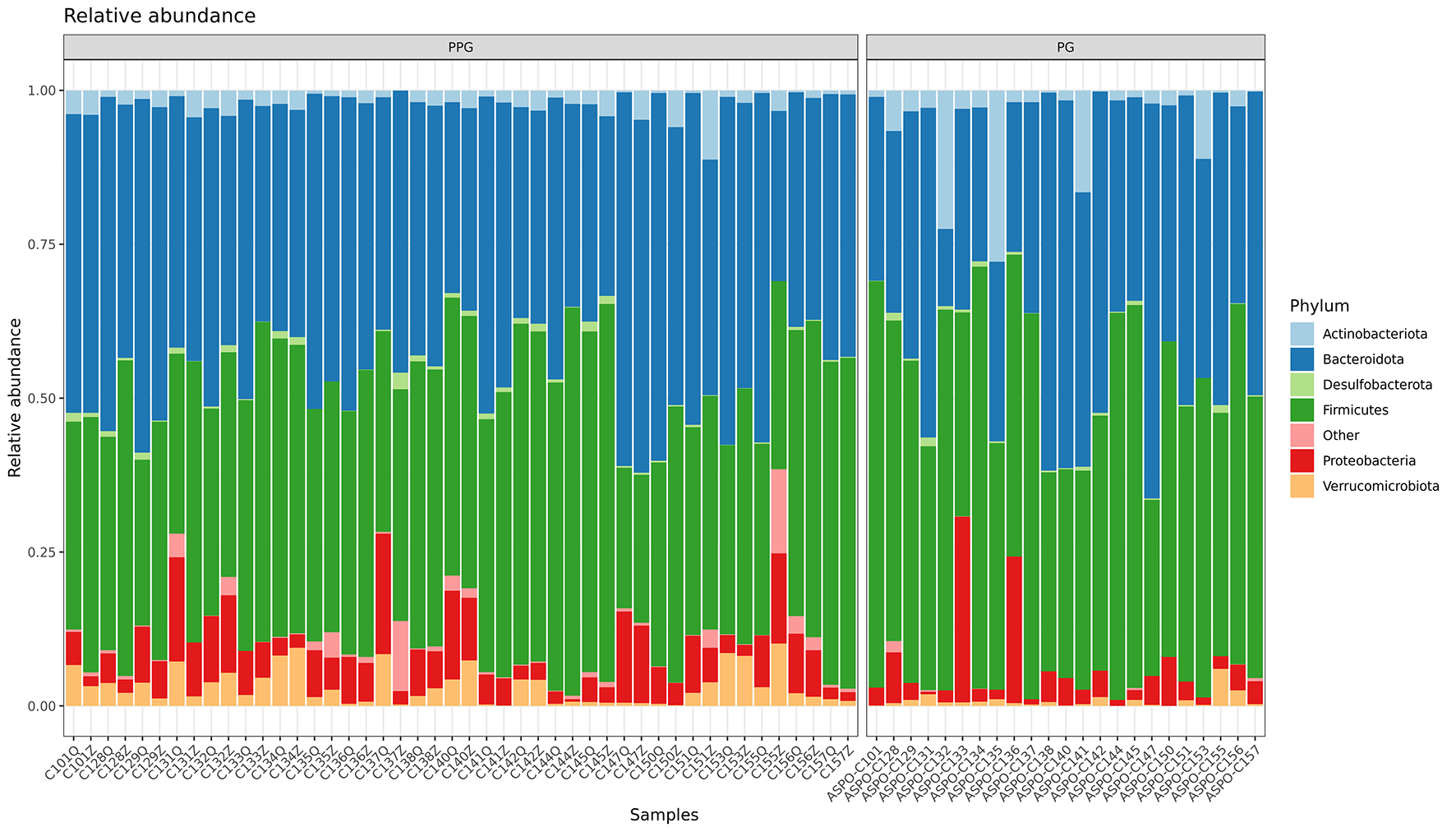

Supplement: Supplementary Figure 3 — Relative abundance and prevalence of the different phyla of the gut microbiota in each subject in the PPG and PG. [file Image_3.TIFF]

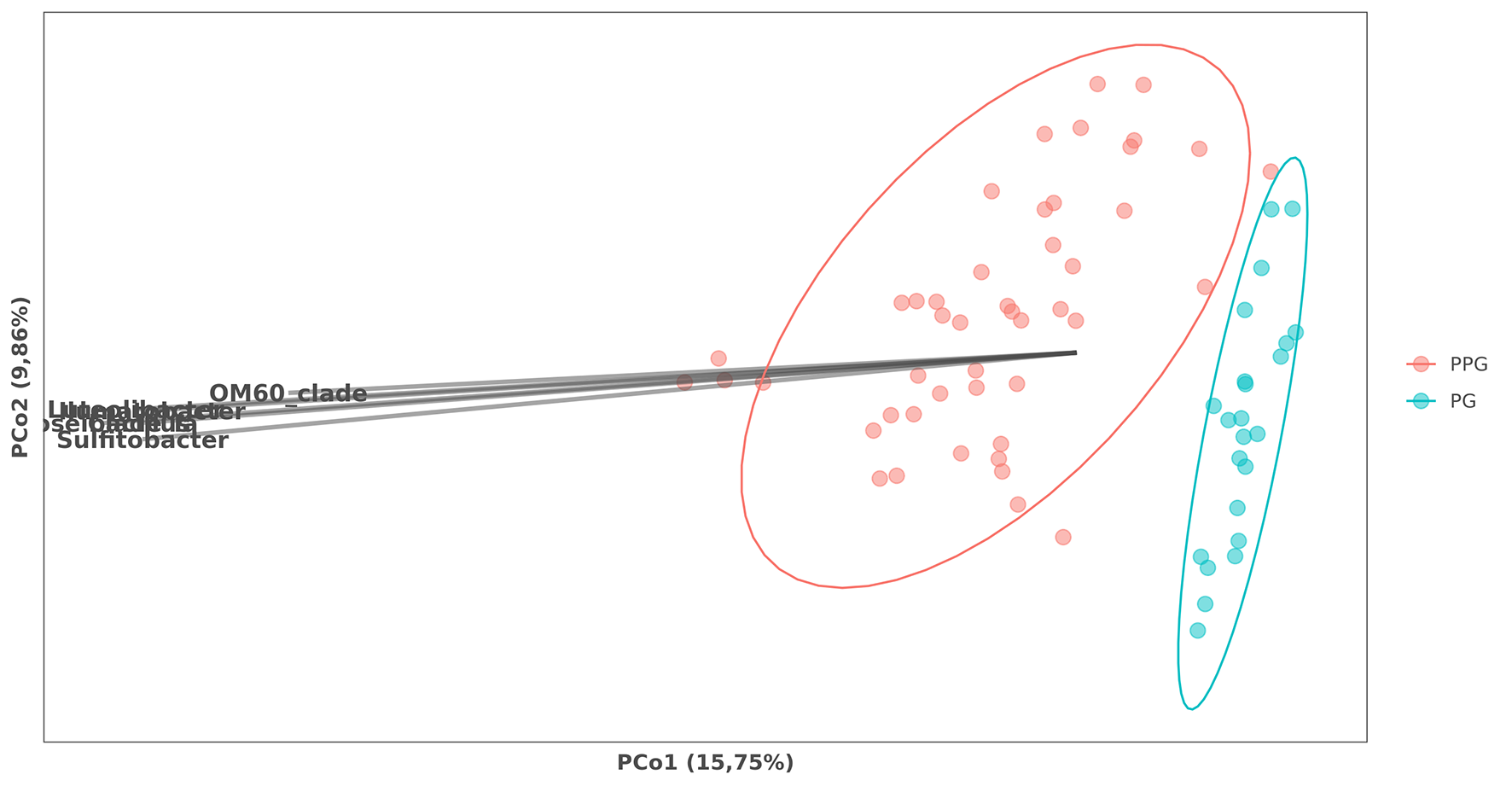

Supplement: Supplementary Figure 4 — Unweighted UniFrac PCoA plots of beta diversity with significant vector correlation by fitting metadata and ASVs vectors on ordination space for bacterial communities. Factors shown had a significant correlation with ordination axes (p < 0.05). [file Image_4.TIFF]
