# Supplementary material for: A Two-Time Point Analysis of Gut Microbiota in the General Population of Buenos Aires and Its Variation Due to Preventive and Compulsory Social Isolation During the COVID-19 Pandemic
Source: Front Microbiol. 2022 Mar 24;13:803121. doi: 10.3389/fmicb.2022.803121 (PMC8988235; doi:10.3389/fmicb.2022.803121)
Supplement: Supplementary File 1 — Pipeline for data analysis used in this study. [file Data_Sheet_1.PDF]

Activate QIIME 2 conda environment [1]

```
conda activate qiime2-2021.2
```

Import FASTQs as QIIME 2 artifact

2016	2020
qiime tools import \ --type SampleData[PairedEndSequencesWithQuality] \ --input-path Manifest2016.csv \ --output-path reads2016_qza/reads.qza \ --input-format PairedEndFastqManifestPhred33	qiime tools import \ --type SampleData[PairedEndSequencesWithQuality] \ --input-path Manifest2020.csv \ --output-path reads2020_qza/reads.qza \ --input-format PairedEndFastqManifestPhred33

Figaro to determine optimal trimming parameters for each group [2]

-a REQUIRED : The length of the amplified sequence target not including primers. Users are required to set this.

2016*	2020
python3 /data/tools/figaro/figaro.py \ -i seqs2016/ \ -o Figaro2016 \ -a 427 \ -f 17 \ -r 20 \ --fileNamingStandard illumina	mkdir Figaro2020<br><br>python3 /data/tools/figaro/figaro.py \ -i seqs2020/ \ -o Figaro2020 \ -a 427 \ -f 17 \ -r 20 \ --fileNamingStandard illumina
**Output**	**Output**
"trimPosition": [271, 213], "maxExpectedError": [5, 5],<br><br>"readRetentionPercent": 77.3, "score": 45.29832530142728	"trimPosition": [265, 219], "maxExpectedError": [2, 2],<br><br>"readRetentionPercent": 80.92, "score": 78.92498738981097

***Since 2016 sequences have different lengths, those reads were trimmed with trimmomatic (CROP:290 , MINLEN:290) before running Figaro**

Running DADA2 workflow

(Plugin version 2021.2.0) [1]

2016	2020
qiime dada2 denoise-paired \ --i-demultiplexed-seqs reads2016_qza/reads.qza \ --p-trim-left-f 17 \ --p-trim-left-r 21 \ --p-trunc-len-f 271 \ --p-trunc-len-r 213 \ --p-max-ee-f 5 \ --p-max-ee-r 5 \ --p-n-threads 30 \ --o-table output_dada2016/table.qza \ --o-representative-sequences output_dada2016/rep-seqs.qza \ --o-denoising-stats output_dada2016/denoising-stats.qza	qiime dada2 denoise-paired \ --i-demultiplexed-seqs reads2020_qza/reads.qza \ --p-trim-left-f 17 \ --p-trim-left-r 21 \ --p-trunc-len-f 265 \ --p-trunc-len-r 219 \ --p-max-ee-f 2 \ --p-max-ee-r 2 \ --p-n-threads 20 \ --o-table output_dada2020/table.qza \ --o-representative-sequences output_dada2020/rep-seqs.qza \ --o-denoising-stats output_dada2020/denoising-stats.qza

Assign taxonomy to ASVs (Silva 138 db)[3]

- **Train supervised learning classifier with RESCRIPt**

To perform Taxonomic classification by qiime feature-classifier classify-sklearn, we train a supervised learning classifier with RESCRIPt package, using V3-V4 primers used in this study and a 99% similarity threshold following the author's tutorial (https://github.com/bokulich-lab/RESCRIPt/).

```
2016 RESCRIPt output file : --o-classifier silva-138-ssu-nr99-341F-805R-classifier.qza

2020 RESCRIPt output file: --o-classifier silva-138-ssu-nr99-337F-805R-classifier.qza
```

- **Run taxonomic classification**

2016	2020
qiime feature-classifier classify-sklearn \ --i-reads output_dada2016/rep-seqs.qza \ --i-classifier Silva/silva-138-ssu-nr99-341F-805R-classif<br>ier.qza \ --output-dir Silva/taxa_2016 \ --verbose	qiime feature-classifier classify-sklearn \ --i-reads output_dada2020/rep-seqs.qza \ --i-classifier Silva/silva-138-ssu-nr99-341F-805R-classif<br>ier.qza \ --output-dir Silva/taxa_2020 \ --verbose

*The performance of the taxonomic classification was assessed by comparing the taxonomic assignments with the top BLASTn hits for certain ASVs.

```
qiime feature-table tabulate-seqs --i-data output_dada2016/rep-seqs.qza
qiime feature-table tabulate-seqs --i-data output_dada2020/rep-seqs.qza
```

Filtering resultant table

- **Filter out rare ASVs**

To exclude ASVs that are likely due to MiSeq bleed-through between runs (reported by Illumina to be 0.1% of reads) we remove all ASVs that have a frequency of less than 0.1% of the mean sample depth.

2016	2020
mean frequency ($\bar{x}$): 60823.86	mean frequency ($\bar{x}$) = 38804.60
SD = 6068.07	SD = 6630.69

```
qiime feature-table filter-features \
   --i-table
/output_dada2016/table.qza \
   --p-min-frequency 61 \
   --p-min-samples 1 \
   --o-filtered-table
output_dada2016/dada_table_filt.qza
```

```
qiime feature-table filter-features \
   --i-table
output_dada2020/table.qza \
   --p-min-frequency 39 \
   --p-min-samples 1 \
   --o-filtered-table
/output_dada2020/dada_table_filt.qza
```

- **Filter out contaminant and unclassified ASVs**

2016	2020

```
qiime taxa filter-table \
   --i-table
output_dada2016/dada_table_filt.qza  \
   --i-taxonomy
Silva/taxa_2016/classification.qza \
   --p-include p__ \
   --p-exclude mitochondria,chloroplast \
   --o-filtered-table
output_dada2016/dada_table_final_Silva.qza
```

```
qiime taxa filter-table \
   --i-table
output_dada2020/dada_table_filt.qza  \
   --i-taxonomy
Silva/taxa_2020/classification.qza \
   --p-include p__ \
   --p-exclude mitochondria,chloroplast \
   --o-filtered-table
output_dada2020/dada_table_final_Silva.qza
```

- **Merge Tables**

```
#Merge otu-table

qiime feature-table merge \
  --i-tables output_dada2016/dada_table_final_Silva.qza \
  --i-tables output_dada2020/dada_table_final_Silva.qza \
  --o-merged-table Silva/Merged_ASPO/dada_table_Merged.qza


#Merge rep-seqs

qiime feature-table merge-seqs \
  --i-data output_dada2016/rep-seqs.qza \
  --i-data output_dada2020/rep-seqs.qza \
  --o-merged-data Silva/Merged_ASPO/rep-seqs_Merged.qza


#Merge taxa

qiime feature-table merge-taxa \
--i-data Silva/taxa_2016/classification.qza \
--i-data Silva/taxa_2020/classification.qza \
--o-merged-data Silva/Merged_ASPO/taxa/classification_Merged.qza
```

Build tree with SEPP QIIME 2 plugin

SEPP (version 4.3.10)[4]

```
qiime fragment-insertion sepp \
   --i-representative-sequences Silva/Merged_ASPO/rep-seqs_Merged.qza \
   --i-reference-database /data/databases/Silva/sepp-refs-silva-128.qza \
   --o-tree Silva/Merged_ASPO/SEPP_Tree/asvs-tree-Silva.qza \
   --o-placements Silva/Merged_ASPO/SEPP_Tree/insertion-placements-Silva.qza \
   --p-threads 20
```

Generate rarefaction curves

Rarefaction curve plotting for all samples to determine if sequencing depth is sufficient (key quality control step).

```
qiime diversity alpha-rarefaction \
   --i-table Silva/Merged_ASPO/dada_table_Merged.qza \
   --p-max-depth 71000 \
```

```
   --p-steps 20 \
   --i-phylogeny Silva/Merged_ASPO/SEPP_Tree/asvs-tree-Silva.qza \
   --m-metadata-file metadata_ASPO_long.tsv \
   --o-visualization Silva/Merged_ASPO/Core_metrics/rarefaction_curves.qzv
```

See the rarefaction curve of each sample individually

```
qiime diversity alpha-rarefaction \
   --i-table Silva/Merged_ASPO/dada_table_Merged.qza \
   --p-max-depth 71000 \
   --p-steps 25 \
   --i-phylogeny Silva/Merged_ASPO/SEPP_Tree/asvs-tree-Silva.qza \
   --o-visualization Silva/Merged_ASPO/Core_metrics/rarefaction_curves_eachsample.qzv
```

Calculating diversity metrics and generating ordination plots

All samples was rarefy to the lowest sample depth before calculate these metrics (29750)

```
qiime diversity core-metrics-phylogenetic \
   --i-table Silva/Merged_ASPO/dada_table_Merged.qza \
   --i-phylogeny  Silva/Merged_ASPO/SEPP_Tree/asvs-tree-Silva.qza \
   --p-sampling-depth 29500 \
   --m-metadata-file metadata_ASPO_long.tsv \
   --p-n-jobs-or-threads 10 \
   --output-dir Silva/Merged_ASPO/Core_metrics/diversity
```

- **Alpha diversity Boxplots**

```
#shannon

qiime diversity alpha-group-significance \
   --i-alpha-diversity Silva/Merged_ASPO/Core_metrics/diversity/shannon_vector.qza \
   --m-metadata-file metadata_ASPO_long.tsv \
   --o-visualization
Silva/Merged_ASPO/Core_metrics/diversity/shannon_compare_groups.qzv


#faith-pd
qiime diversity alpha-group-significance \
  --i-alpha-diversity Silva/Merged_ASPO/Core_metrics/diversity/faith_pd_vector.qza \
  --m-metadata-file metadata_ASPO_long.tsv \
  --o-visualization
Silva/Merged_ASPO/Core_metrics/diversity/faith-pd-group-significance.qzv



#observed-features
```

```
qiime diversity alpha-group-significance \
  --i-alpha-diversity
Silva/Merged_ASPO/Core_metrics/diversity/observed_features_vector.qza \
  --m-metadata-file metadata_ASPO_long.tsv \
  --o-visualization
Silva/Merged_ASPO/Core_metrics/diversity/observed-features-group-significance.qzv
```

- **Generate stacked barchart of taxa relative abundances**

```
qiime taxa barplot \
   --i-table Silva/Merged_ASPO/dada_table_Merged.qza \
   --i-taxonomy Silva//Merged_ASPO/taxa/classification_Merged.qza \
   --m-metadata-file  metadata_ASPO_long.tsv \
   --o-visualization Silva/Merged_ASPO/Core_metrics/taxa/taxa_barplot.qzv
```

read_qza and qza_to_phyloseq functions from qiime2R package was used in order to import qza to R

Rarefaction, Alpha and beta diversity plots were generated by using ggplot function from ggplot2 package

Taxa barplot was generated by using plot_composition functions from microbiome package

ADONIS [5]

https://CRAN.R-project.org/package=vegan) implemented in QIIME2 (q2-diversity plugin 2021.2.0)

```
##Unweighted_unifrac_distance Adonis


qiime diversity adonis \
   --i-distance-matrix
Silva/Merged_ASPO/Core_metrics/diversity2/unweighted_unifrac_distance_matrix.qza \
    --m-metadata-file metadata_ASPO_long.tsv \
    --p-formula 'Description+PurificationKit+SubjectID' \
    --p-permutations 1000\
    --p-n-jobs 16 \
    --o-visualization
Silva/Merged_ASPO/Core_metrics/diversity2/Adonis/unweighted_unifrac_Description_Ki
t_ID_significance.qzv

##weighted_unifrac_distance Adonis

qiime diversity adonis \
   --i-distance-matrix
Silva/Merged_ASPO/Core_metrics/diversity2/weighted_unifrac_distance_matrix.qza \
    --m-metadata-file metadata_ASPO_long.tsv \
    --p-formula 'Description+PurificationKit+SubjectID' \
    --p-permutations 1000\
```

```
    --p-n-jobs 16 \
    --o-visualization
Silva/Merged_ASPO/Core_metrics/diversity2/Adonis/weighted_unifrac_Description__Kit
_ID_significance.qzv
```

Identifying differentially abundant features with ANCOM

```
#Pseudocount addition (1) all features must have nonzero abundances

qiime composition add-pseudocount \
   --i-table Silva/Merged_ASPO/dada_table_Merged.qza\
   --p-pseudocount 1 \
   --o-composition-table Silva/Merged_ASPO/dada_table_Merged_pseudocount.qza

qiime composition ancom \
   --i-table Silva/Merged_ASPO/dada_table_Merged_pseudocount.qza \
   --m-metadata-file metadata_ASPO_long.tsv \
   --m-metadata-column Description \
   --output-dir Silva/Merged_ASPO/Ancom_output_Description




#ANCOM_Level_2

qiime taxa collapse \
  --i-table Silva/Merged_ASPO/dada_table_Merged.qza \
  --i-taxonomy Silva/Merged_ASPO/taxa/classification_Merged.qza \
  --p-level 2 \
  --o-collapsed-table Silva/Merged_ASPO/dada_table_Merged_L2.qza

qiime composition add-pseudocount \
  --i-table Silva/Merged_ASPO/dada_table_Merged_L2.qza \
  --o-composition-table Silva/Merged_ASPO/dada_table_Merged_L2_pseudocount.qza

qiime composition ancom \
  --i-table Silva/Merged_ASPO/dada_table_Merged_L2_pseudocount.qza \
  --m-metadata-file metadata_ASPO_long.tsv \
  --m-metadata-column Description \
  --o-visualization Silva/Merged_ASPO/Ancom/L2-ancom-Description.qzv



#ANCOM_Level_6

qiime taxa collapse \
  --i-table Silva/Merged_ASPO/dada_table_Merged.qza \
  --i-taxonomy Silva/Merged_ASPO/taxa/classification_Merged.qza \
```

```
  --p-level 6 \
  --o-collapsed-table tableSilva/Merged_ASPO/dada_table_Merged_L6.qza

qiime composition add-pseudocount \
  --i-table Silva/Merged_ASPO/dada_table_Merged_L6.qza \
  --o-composition-table Silva/Merged_ASPO/dada_table_Merged_L6_pseudocount.qza

qiime composition ancom \
  --i-table Silva/Merged_ASPO/dada_table_Merged_L6_pseudocount.qza \
  --m-metadata-file metadata_ASPO_long.tsv \
  --m-metadata-column Description \
  --o-visualization Silva/Merged_ASPO/Ancom/L6-ancom-Description.qzv
```

Volcano plots were generated based on the qiime2 output (L6-ancom-Description.qzv) by the ggplot function from ggplot2 package

Pictrust2 [6]

Phylogenetic Investigation of Communities by Reconstruction of Unobserved States (PICRUSt version 2.4.1), implemented in QIIME2

```
#Pictrust2

qiime picrust2 full-pipeline \
   --i-table Silva/Merged_ASPO/dada_table_Merged.qza \
   --i-seq Silva/Merged_ASPO/rep-seqs_Merged.qza \
   --o-pathway-abundance Silva/Merged_ASPO/Picrust/Prediction_MetaCyc.qza \
   --o-ko-metagenome Silva/Merged_ASPO/Picrust/Prediction_EC.qza \
   --o-ec-metagenome Silva/Merged_ASPO/Picrust/Prediction_OK.qza \
   --p-threads 10 \
   --p-hsp-method mp \
   --p-max-nsti 2 \
   --p-edge-exponent 0 \
   --verbose
```

Calculating diversity metrics for Picrust outputs

2586471

```
#Pictrust2 diversity core-metrics

qiime diversity core-metrics \
```

```
  --i-table Silva/Merged_ASPO/Picrust/Prediction_MetaCyc.qza \
  --p-sampling-depth 2586471 \
  --m-metadata-file metadata_ASPO_long.tsv \
  --output-dir Silva/Merged_ASPO/Picrust/Predict.Cyc_metrics  \
  --p-n-jobs 10

qiime diversity alpha-group-significance \
  --i-alpha-diversity
ASPO_long/Silva/Merged_ASPO/Picrust/Predict.Cyc_metrics2/shannon_vector.qza \
  --m-metadata-file ASPO_long/metadata_ASPO_long.tsv \
  --o-visualization
ASPO_long/Silva/Merged_ASPO/Picrust/Predict.Cyc_metrics2/shannon_vector.qzv


qiime diversity alpha-group-significance \
  --i-alpha-diversity
ASPO_long/Silva/Merged_ASPO/Picrust/Predict.Cyc_metrics2/evenness_vector.qza \
  --m-metadata-file ASPO_long/metadata_ASPO_long.tsv \
  --o-visualization
ASPO_long/Silva/Merged_ASPO/Picrust/Predict.Cyc_metrics2/evenness_vector.qzv

qiime diversity alpha-group-significance \
  --i-alpha-diversity
ASPO_long/Silva/Merged_ASPO/Picrust/Predict.Cyc_metrics2/observed_features_vec
tor.qza \
  --m-metadata-file ASPO_long/metadata_ASPO_long.tsv \
  --o-visualization
ASPO_long/Silva/Merged_ASPO/Picrust/Predict.Cyc_metrics2/observed_features_vec
tor.qzv
```

Identifying differentially abundant Metabolic features with ANCOM

```
qiime composition add-pseudocount \
  --i-table Silva/Merged_ASPO/Picrust/Prediction_MetaCyc.qza \
  --o-composition-table
Silva/Merged_ASPO/Picrust/Compositional/comp-table-Groups_Cyc.qza


qiime composition ancom \
  --i-table Silva/Merged_ASPO/Picrust/Compositional/comp-table-Groups_Cyc.qza
\
  --m-metadata-file metadata_ASPO_long.tsv \
  --m-metadata-column Description \
  --o-visualization
Silva/Merged_ASPO/Picrust/Compositional/Ancom-Description_Cyc.qzv
```

Volcano plots were generated based on the qiime2 output (Ancom-Description_Cyc.qzv) by the

ggplot function from ggplot2 package

Core-features: Identify core features in table

Core features was determined by core_members function from microbiome R package

```
core_m <- core_members(ps.sub,
                            detection = 0.001,
                            prevalence = 50/100)
```

In order to calculate the contribution of each genera with the unweighted UniFrac PCoA envfit function from vegan package was used.

BIBLIOGRAPHY

1. Callahan BJ, McMurdie PJ, Rosen MJ, Han AW, Johnson AJA, Holmes SP. DADA2: High-resolution sample inference from Illumina amplicon data. Nature Methods. 2016. pp. 581–583. doi:10.1038/nmeth.3869
2. Weinstein MM, Prem A, Jin M, Tang S, Bhasin JM. FIGARO: An efficient and objective tool for optimizing microbiome rRNA gene trimming parameters. doi:10.1101/610394
3. Quast C, Pruesse E, Yilmaz P, Gerken J, Schweer T, Yarza P, et al. The SILVA ribosomal RNA gene database project: improved data processing and web-based tools. Nucleic Acids Research. 2012. pp. D590–D596. doi:10.1093/nar/gks1219
4. Mirarab S, Nguyen N, Warnow T. SEPP: SATé-Enabled Phylogenetic Placement. Biocomputing 2012. 2011. doi:10.1142/9789814366496_0024
5. Anderson MJ. A new method for non-parametric multivariate analysis of variance. Austral Ecology. 2001. pp. 32–46. doi:10.1111/j.1442-9993.2001.01070.pp.x
6. Douglas GM, Maffei VJ, Zaneveld JR, Yurgel SN, Brown JR, Taylor CM, et al. PICRUSt2 for prediction of metagenome functions. Nat Biotechnol. 2020;38: 685–688.
